# Hemophagocytic Lymphohistiocytosis Complicating T-Cell Lymphoma in a Patient with HIV Infection

**DOI:** 10.1155/2013/687260

**Published:** 2013-08-31

**Authors:** Marc Uemura, Richard Huynh, Allen Kuo, Fernando Antelo, Robert Deiss, James Yeh

**Affiliations:** ^1^Department of Internal Medicine, Harbor-UCLA Medical Center, Torrance, CA 90502, USA; ^2^Department of Pathology, Harbor-UCLA Medical Center, Torrance, CA 90502, USA; ^3^Division of Infectious Diseases, Harbor-UCLA Medical Center, Torrance, CA 90502, USA

## Abstract

Hemophagocytic lymphohistiocytosis (HLH), while uncommon, may be a devastating complication of lymphoma and/or human immunodeficiency virus (HIV) infection. While several of the diagnostic criteria for HLH are relatively nonspecific, particularly in the setting of a systemic inflammatory response, more diagnostic specificity may be achieved with marked elevations in serum ferritin (e.g., >100,000 ng/mL). Increased suspicion of HLH, particularly in the setting of persistent, unexplained fevers, pancytopenia, and transaminitis, should prompt consideration of HLH. Earlier diagnosis and initiation of therapy have the potential to alter the natural history and poor prognosis of this disorder. We present a patient with HIV infection who developed relapsed T-cell lymphoma complicated by hemophagocytic lymphohistiocytosis.

## 1. Introduction

Hemophagocytic lymphohistiocytosis (HLH) is an uncommon, often life-threatening clinical syndrome characterized by fever, hepatosplenomegaly, abnormal transaminases, hyperferritinemia, coagulopathy, and pancytopenia [1]. This syndrome may result from cytotoxic immune dysregulation leading to abnormal T-lymphocyte and macrophage activation and subsequent inflammatory cytokine production [2]. Lymphocytic infiltration may occur in many visceral and hematopoietic organs, including the bone marrow, lymph nodes, spleen, and liver. Biopsies of these sites may show macrophages phagocytosing erythrocytes, leukocytes and platelets [3]. This systemic inflammatory process ultimately results in multiorgan dysfunction.

Primary forms of HLH are due to hereditary abnormalities of immune function, including mutations of the perforin gene [4]. Secondary forms have been associated with infections, collagen vascular diseases, and malignancies, particularly lymphomas and leukemias [5]. 

The constellation of HLH, T-cell lymphoma, and HIV infection appears to be a rare event, with only one case report identified in our search of the medical literature [6]. We report a case of HLH occurring in the setting of relapsed HIV-associated T-cell lymphoma. 

## 2. Case Presentation

**Figure 1:**
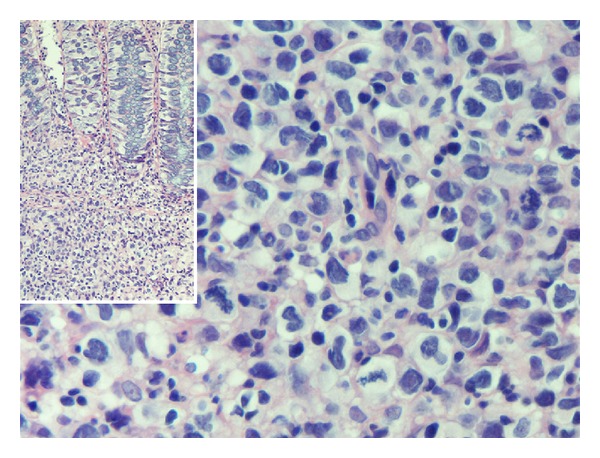
Light micrograph of hematoxylin and eosin stained colon biopsies showing high grade T-cell lymphoma cells deep within the glands.

A 37-year-old Hispanic male initially underwent colonoscopy at an outside facility for lower gastrointestinal bleed and had a biopsy that was suspicious for lymphoma. He was subsequently evaluated at our hospital, where a repeat colonoscopy with biopsy of an ascending colon lesion revealed high-grade T-cell lymphoma (Figure 1). Staging studies, including bone marrow biopsy and cerebrospinal fluid cytology, were negative for lymphoma. Computed tomography scans revealed splenomegaly, pulmonary infiltrates, and an ascending colon lesion. A PET scan showed uptake in the colon, right lower lung lobe, and spleen. A bronchoscopy did not identify lymphomatous or infectious processes. He was deemed to have stage IIIE versus stage IV disease.

The patient was diagnosed with HIV infection four years prior to presentation and had been compliant with highly active antiretroviral therapy, including emtricitabine, darunavir, and ritonavir. At the time he was diagnosed with lymphoma, his CD4 count was 123 per mm^3^, and his viral load was undetectable. The patient's medical history was otherwise unremarkable.

The patient completed six cycles of chemotherapy with cyclophosphamide, doxorubicin, vincristine, and prednisone (CHOP). Four weeks following his last cycle, he was admitted for weakness, dizziness, dyspnea, and melena. He denied abdominal pain, cough, or constitutional symptoms. On examination, he was febrile, tachycardic, and hypotensive. Cardiac, pulmonary, skin, and neurologic exams were otherwise unremarkable; palpation did not reveal lymphadenopathy or hepatosplenomegaly.

Admission laboratory studies revealed hemoglobin of 6.8 g/dL (13.8–16.9 g/dL), leukocyte count of 3.0 × 10^9^/L (4.0–10.0 × 10^9^/L), and platelet count of 102 × 10^9^/L (150–420 × 10^9^/L). His aspartate aminotransferase was 102 U/L (15–41 U/L); alanine aminotransferase, 44 U/L (10–40 U/L); and alkaline phosphatase, 142 U/L (38–126 U/L). Other laboratory abnormalities included elevations in triglycerides (312 mg/dL) and lactate dehydrogenase (1,605 U/L). Additionally, his ferritin was markedly elevated at 112,725 ng/mL (23.9–336.2 ng/mL). A serum ferritin level checked three weeks prior as part of a workup for microcytic anemia was 153 ng/mL. A repeat CD4 count on admission was 2 per mm^3^.

The patient was admitted for severe sepsis and received early goal-directed therapy and empiric broad-spectrum antibiotics with cefepime, metronidazole, and vancomycin. Antiretroviral therapy was continued. Despite these measures, he remained persistently febrile and hypotensive. Itraconazole was added to his antimicrobial regimen, without improvement. A broad infectious workup including blood, urine, respiratory, fungal, and mycobacterial cultures was unrevealing. Additional studies for histoplasma and coccidioidomycosis were negative. The patient's serum procalcitonin level was moderately elevated at 6.08 ng/mL.

**Figure 2:**
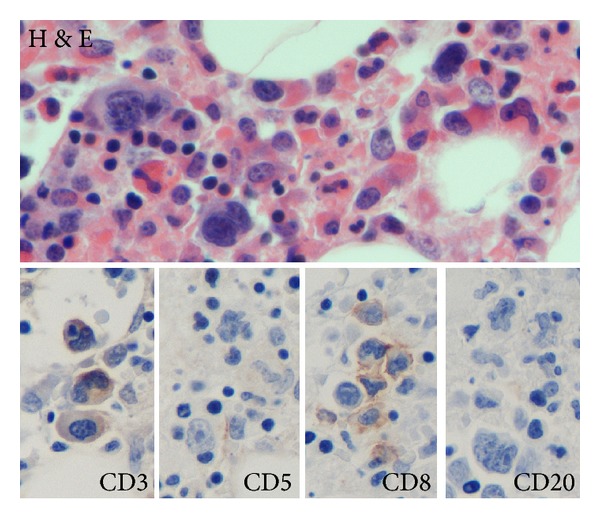
Light micrograph of hematoxylin and eosin stained bone marrow section showing lymphoma cells with atypical nuclei. Immunohistochemistry (CD3, CD5, and CD8) illustrates the T-cell origin of the lymphoma. CD20 was also noted to be weakly positive.

Given the patient's persistent pancytopenia, a bone marrow biopsy was performed which showed scattered, large atypical cells with irregular nuclei with multiple nucleoli (Figure 2). Immunohistochemical staining of these cells was positive for CD3, CD5, and CD8, consistent with intramedullary relapse of T-cell lymphoma. Clot specimens from the bone marrow aspirate contained rare cells suspicious for hemophagocytosis. The bone marrow specimen, along with the constellation of fever, splenomegaly, pancytopenia, hypertriglyceridemia, and elevated ferritin, fulfilled the diagnostic criteria for HLH.

The patient was started on cyclosporine and dexamethasone as per the HLH-2004 protocol. Etoposide and intravenous immunoglobulin were omitted due to hepatic and renal dysfunction, respectively. Prophylactic doses of acyclovir and atovaquone were added to continued antimicrobial therapy for ongoing sepsis. The patient also received intense transfusion support. 

The patient initially responded favorably to this therapy, becoming afebrile with subjective improvement. A repeat procalcitonin remained elevated at 4.10 ng/mL. However, four days after beginning therapy for HLH, the patient experienced a seizure, and an emergent CT scan showed a spontaneous brainstem hemorrhage. Brain death was declared shortly thereafter. 

## 3. Discussion

HLH represents a syndrome of pathologic immune activation of T-lymphocytes and macrophages leading to uncontrolled phagocytosis of erythrocytes, leukocytes, and platelets throughout the reticuloendothelial system. HLH diagnosis may be established by demonstrating at least five of eight established clinical criteria (Table 1). 

While hyperferritinemia is not specific and can occur in a wide variety of inflammatory disorders, very few disorders are associated with ferritin levels exceeding 10,000 ng/mL. Among these, adult Still's disease, histiocytic malignancies, and HLH have a reported association with ferritin levels >10,000 ng/mL. However, ferritin levels >60,000 ng/mL have almost exclusively been reported with HLH [7]. Our patient displayed a ferritin level >100,000 ng/mL, which we deemed a major diagnostic clue for HLH. In evaluating patients with such extreme ferritin elevations, one should strongly consider HLH.

More specific biomarkers may help distinguish the inflammation associated with HLH from other conditions. The procalcitonin (PCT) assay is an emerging marker of systemic inflammation. Marked PCT elevations have been most consistently identified in bacterial sepsis, but may occur with noninfectious processes such as trauma, burns, and pancreatitis [8–10]. Our case report underscores the association of HLH with elevated PCT levels, as has been previously reported [11].

HLH has a well-documented association with infections. Virus-associated HLH was first identified in 1979 in a cohort of patients infected with herpes group viruses and adenoviruses [12]. Since then, other viral subtypes have been associated with HLH, including human herpesvirus-8, Epstein-Barr virus, and cytomegalovirus. 

Cases of HLH occurring in the setting of HIV infection have been attributed both to this virus and to other infections or malignancies associated with this infection [13–16]. As clinical features of HLH may resemble opportunistic infections associated with HIV, it is possible that HLH may be underrecognized in HIV-infected individuals [13]. 

Although not fully elucidated, the molecular pathogenesis of HLH appears to be linked to abnormal T-lymphocyte cytotoxicity [2, 17]. Murine models of familial HLH have identified a mutation of a *perforin* gene that encodes a pore-forming protein in the cell membranes of T-lymphocytes and NK cells; this protein regulates apoptosis [4]. *Perforin* mutations may result in cytotoxic abnormalities that compromise normal immune function. Similarly, infections, malignancies, and other inflammatory conditions may cause genetic damage leading to uncontrolled immune activation and HLH. 

The relationship between HLH, HIV, and T-cell lymphomas is not well understood as few such cases exist. HIV infection has been associated with a markedly increased relative risk of malignancies, including lymphoma [13]. While the overwhelming majority of HIV-associated lymphomas are of B cell origin, T-cell lymphomas are markedly less common with fewer than 100 cases reported [18]. A retrospective study of an AIDS-lymphoma registry revealed that T-cell lymphomas comprised only three percent of all AIDS-associated lymphomas [19]. 

The Epstein-Barr virus has also been associated with both lymphomas and HLH. EBV infection has previously been postulated to contribute to HLH by upregulating viral product LMP-1, which activates and stimulates macrophages to phagocytose hematopoietic cells. Proinflammatory cytokines TNF*α* and IFN*γ* produced by EBV-infected T-lymphocytes also contribute to the pathogenesis [5, 17]. 

In our patient, we suspect that both HIV infection and the T-cell lymphoma likely contributed to the development of HLH, possibly through mechanisms related to EBV infection. Given the patient's history of HIV infection, viral incorporation into lymphocyte DNA, combined with the overwhelming trophic response of the relapsed T-cell lymphoma, could have overstimulated the immune system, leading to HLH. 

Because so few cases and such a broad range of triggers for HLH exist, a standard therapeutic regimen for this syndrome is difficult to develop. Current management of HLH involves immunosuppressive and cytotoxic therapy combined with antimicrobial prophylaxis, followed, in selected cases, by allogeneic stem cell transplantation. At our institution, patients most commonly receive dexamethasone, cyclosporine, and etoposide, as specified by the HLH-2004 protocol.

We believe that increased recognition of HLH among clinicians will facilitate more research into the pathogenesis of HLH, including the interaction between HIV infection and lymphoma, which will hopefully translate into more effective therapies for this devastating disorder. 

## Figures and Tables

**Table 1:** Diagnostic criteria for hemophagocytic lymphohistiocytosis.

(A) Molecular diagnosis consistent with HLH	(B) Five of the eight criteria listed below
Pathologic mutations of PRF1, UNC13D, Munc18-2, Rab-27A, STX11, SH2D1A, or BIRC4	(1) Fever > 38.5°C (2) Splenomegaly (3) Cytopenias (affecting at least 2 of 3 lineages in the peripheral blood) (a) Hemoglobin < 9 g/dL (b) Platelets < 100 × 10^3^/mL (c) Neutrophils < 1 × 10^3^/mL (4) Hypertriglyceridemia (fasting > 265 mg/dL) and/or hypofibrinogenemia (< 150 mg/dL) (5) Hemophagocytosis in bone marrow, spleen, lymph nodes, or liver (6) Low or absent NK cell activity (7) Ferritin > 500 ng/mL (8) Elevated sCD25
